# Four-dimensional dosimetry validation and study in lung radiotherapy using deformable image registration and Monte Carlo techniques

**DOI:** 10.1186/1748-717X-5-45

**Published:** 2010-05-29

**Authors:** Tzung-Chi Huang, Ji-An Liang, Thomas Dilling, Tung-Hsin Wu, Geoffrey Zhang

**Affiliations:** 1 Department of Biomedical Imaging and Radiological Science, China Medical University, Taiwan; 2 Radiation Oncology, China Medical University Hospital, Taiwan; 3 Radiation Oncology, Moffitt Cancer Center, Tampa, Florida, USA; 4 Department of Biomedical Imaging and Radiological Sciences, National Yang Ming University, Taiwan

## Abstract

Thoracic cancer treatment presents dosimetric difficulties due to respiratory motion and lung inhomogeneity. Monte Carlo and deformable image registration techniques have been proposed to be used in four-dimensional (4D) dose calculations to overcome the difficulties. This study validates the 4D Monte Carlo dosimetry with measurement, compares 4D dosimetry of different tumor sizes and tumor motion ranges, and demonstrates differences of dose-volume histograms (DVH) with the number of respiratory phases that are included in 4D dosimetry. BEAMnrc was used in dose calculations while an optical flow algorithm was used in deformable image registration and dose mapping. Calculated and measured doses of a moving phantom agreed within 3% at the center of the moving gross tumor volumes (GTV). 4D CT image sets of lung cancer cases were used in the analysis of 4D dosimetry. For a small tumor (12.5 cm^3^) with motion range of 1.5 cm, reduced tumor volume coverage was observed in the 4D dose with a beam margin of 1 cm. For large tumors and tumors with small motion range (around 1 cm), the 4D dosimetry did not differ appreciably from the static plans. The dose-volume histogram (DVH) analysis shows that the inclusion of only extreme respiratory phases in 4D dosimetry is a reasonable approximation of all-phase inclusion for lung cancer cases similar to the ones studied, which reduces the calculation in 4D dosimetry.

## Introduction

Monte Carlo simulation is the most accurate radiation dose calculation algorithm in radiotherapy [1,2]. With the advent of increasingly fast computers and optimized computational algorithms, Monte Carlo methods promise to become the primary dose calculation methodology in future treatment planning systems [3-6]. Thoracic tumor motion could introduce discrepancies between the dose as planned and actually delivered, both to the tumor and the surrounding normal lung [7]. Incorporating Monte Carlo methods into 4-dimensional (4D, 3 spatial dimensions plus time) dosimetry and treatment planning yields the most accurate dose calculations for thoracic tumor treatments [8,9].

To generate a 4D Monte Carlo dose calculation, it is necessary to calculate the dose on CT image sets derived from different time points across the respiratory cycle. These can then be fused together to calculate cumulative doses. Deformable image registration is an integral part of this process. It provides a voxel-to-voxel link between the multiple respiratory phases of a 4D CT image set so that the dose distribution on each phase can correctly be summed to give a path-integrated average dose distribution [10,11]. Deformable image registration across the various phases of a 4D CT image set has become a new focus of study [10,11].

In this study, 4D Monte Carlo dosimetry was presented. The 4D cumulative point dose in a moving phantom was compared with measurement. Clinical lung cancer cases were studied with the goal of determining under which conditions 4D Monte Carlo dosimetry likely differs from a static plan and how many respiratory phases are necessary to be included in 4D dose calculation.

## Materials and methods

### CT-Based Treatment Planning

A total of four CT simulation image sets were used in this study. Two were performed on actual patients. Two lung cancer patients underwent 4D CT scanning (Case 1 and Case 2). These 4D CT data sets were comprised of a total of 10 CT scans per patient, taken at equally-spaced intervals across the entire respiratory cycle (phase-based sorting in 4D CT reconstruction). There were 93 and 94 slices in each respiratory phase of the two 4D CT cases, respectively. The GTV moved about 1.5 cm during the respiratory cycle in Case 1 and 1.0 cm in Case 2, predominantly in the SI direction. The GTV volume for Case 1 was 12.5 cm^3 ^(about 3 cm in diameter) while for Case 2 it was 159.1 cm^3 ^(about 7 cm in diameter). For the last two cases, 4D CT image sets were generated from a moving phantom with two different motion ranges, to compare the 4D cumulative doses with actual measurements. The 4D scans of the moving phantom contained 90 slices in each of the ten respiratory phases. All 4D CT imaging was performed on a 16-slice Big Bore CT scanner (Philips Medical Systems, Andover, MA). The transaxial slice resolution was about 1 mm × 1 mm and the slice thickness was 3 mm for all scans.

**Figure 1:**
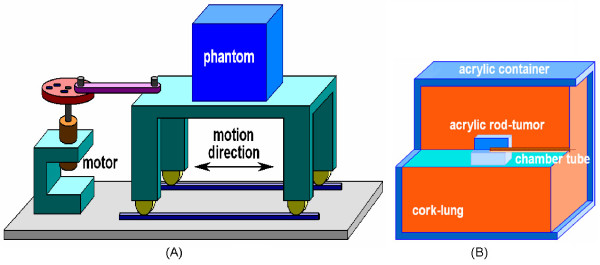
**A. The moving phantom was controlled by a motor with variable rotation frequency**. The rotation wheel had variably-spaced holes in the radial direction which controlled the motion range. B. The phantom had cork placed within an acrylic container to simulate lungs. An acrylic rod was placed within the cork to simulate a tumor. An ion chamber was inserted into the acrylic rod to measure the point dose.

The moving phantom was custom-designed (Figure 1). Phantom motion was controlled by a motor with adjustable rotational frequency. A rotating wheel connected to the motor. The wheel contained holes at various distances from the axis of rotation, which thereby determined the magnitude of the range of the sinusoid motion of the phantom, which is the only motion pattern the table can perform. The phantom container was made of acrylic. Cork blocks with density of 0.26 g/cm^3 ^were placed inside the acrylic container to simulate normal lung. An acrylic rod of 3 × 3 × 2 cm^3 ^was placed in the center of the cork blocks to simulate a tumor. The center of this rod contained a 0.04 cc Scanditronix CC04 ion chamber (active length 3.6 mm, inner radius 2 mm) to measure the point dose. The motion range was set to 1.5 (Case 3) or 3 cm (Case 4) at a frequency of about 18 cycles per minute to simulate respiration. The same motion pattern was used during both the 4D CT scan and treatment delivery.

A treatment plan was generated for each of the four CT data sets. Simple 3D-conformal plans were utilized. All the plans were calculated for a Varian Clinac 2100EX linear accelerator (Varian Medical Systems, Palo Alto, CA). Photon beams of 6 MV in energy were used. The margin from gross tumor volume (GTV) to block edge is 0.5 cm (Case 2) and 1 cm (Case 1, 3 and 4). MLC was used for the conformal plans in Case 1 and 2. Open 5 × 5 cm^2 ^beams were used in the phantom study cases due to the regular shape of the acrylic rod which simulated the GTV.

For Case 1 and Case 2, the tumors were contoured on the maximum inspiration phase of the respective 4D CT image sets and the isocenters were set accordingly. A 3D plan was then generated for each patient. For Case 1, a wedged 3-beam 3D plan was created. A wedged two-field 3D-conformal plan was designed for Case 2. The respective treatment plans were then copied over from the maximum inspiration scan to each of the other nine phases of the CT scan for that patient. A Monte Carlo simulation was used to calculate the dose distribution on each phase. The dose distributions from all other phases were mapped to the maximum inspiration phase using deformation matrices generated via deformable image registration between all the other phase and the maximum inspiration phase. A 4D cumulative dose distribution was created from an equally-weighted average of the dose distributions. This 4D Monte Carlo dosimetry method was applied to the two cases over all ten phases (*vide infra*). A dose-volume-histogram (DVH) was obtained for each of the respiration phases and the 4D integrated DVH was obtained from the 4D cumulative dose distribution.

For the moving phantom cases, a lateral-opposed 2-beam plan was designed to cover the simulated tumor during the maximum inspiration phase. These beams were copied to the nine other phases of CT scans and the doses were calculated using Monte Carlo methods (*vide infra*). The 4D cumulative doses were generated.

Table 1 lists the tumor sizes, motion ranges and beam margins for all the cases studied. The beam margins are purposely set smaller than the motion ranges to gauge the coverage loss effects.

**Table 1:** Relevant parameters in the cases studied

Case	1	2	3	4
Subject	Patient	Patient	Phantom	Phantom

GTV size/cc	12.5	159.1	18.0	18.0

Motion range/cm	1.5	1.0	1.5	3.0

Margin/cm	1.0	0.5	1.0	1.0

### Monte Carlo Dose Calculation

BEAMnrc [1] was used to simulate the linear accelerator. This is a Monte Carlo simulation application based on EGSnrc [12], a software package designed for Monte Carlo simulation of coupled electron-photon transport. The simulated incident electron beam bombarding the tungsten target was a 6 MeV pencil beam with a 2-dimensional Gaussian distribution of full width at half maximum (FWHM) of 0.1 cm [1,12]. For each treatment beam, the linear accelerator was simulated to generate a phase-space file containing information about each particle exiting the treatment head of the machine, as it existed at 60 cm from the electron source. The percentage depth dose curves and profiles in a water phantom from Monte Carlo simulations were matched with the measured data within 2% for most of the low gradient dose regions and slightly over 2% at the shoulders of one of the profiles. In regions of build-up or penumbra, the distance between calculated and measured curves was within1 mm.

Another EGSnrc based software, DOSXYZnrc [13], was used for dose calculations in the patient/phantom through the various respiratory phases. Additionally, CT-to-phantom converter code, ctcreate [14], was used to convert the patient/phantom CT image data to CT phantom data that DOSXYZnrc could use. For the patient cases (Case 1 & 2), AIR, LUNG, ICRUTISSUE and ICRPBONE were used for air, lung tissue, soft tissue and bone media respectively based on their CT number ranges, while for the phantom cases (Case 3 & 4), AIR, LUNG and PMMA were used for air, cork and acrylic respectively. Dosimetrically, cork is equivalent to lung tissues [15,16]. The dose grid size used for this study was 2 × 2 × 3 mm^3^, which is coarser than the CT image resolution of 1 × 1 × 3 mm^3^. Each CT slice was therefore sub-sampled from 512 × 512 pixels to 256 × 256 pixels to match the Monte Carlo dose grid size before the CT-to-phantom conversion. The phase-space files were then used as the particle source to calculate the dose distribution for each respiratory phase in the patients and phantom. In order to achieve acceptable statistical uncertainties in target volume (about 1%), the particles stored in the phase space files were recycled 4 times. No specific variance reduction technique was applied. The cutoff energies for electrons (ECUT) and for photons (PCUT) were 0.7 and 0.01 MeV respectively. Dose calculation for one respiratory phase took about 20 hours of CPU time on a 2.66 GHz single-processor personal computer with 2 GB RAM, running Linux.

### Deformable Image Registration

The optical flow method of deformable image registration was then applied to calculate the deformation matrices between the CT images from the different respiratory phases. These matrices were used to map the dose distributions from the various respiratory phases to an average integral dose. The 3D optical flow program was based upon the 2D Horn and Schunck algorithm [11,17].

For typical 4D CT image sets with a sub-sampled slice resolution of 2 × 2 mm^2^/pixel, each deformable image registration required about three minutes on a personal computer with a single 2.66 GHz CPU and 4 GB RAM. Thus, for a respiratory cycle divided into 10 phases, about half an hour was required to calculate all the deformation matrixes.

## Results

### Moving Phantom Study

Absolute dose was used in the 4D dosimetry of the moving phantom by normalizing the dose matrix to the reference dose which was the maximum value of the central depth dose of a 10 × 10 cm^2 ^field at 100 cm of source to surface distance (SSD). This absolute dose conversion assumed that the Monte Carlo calculated reference dose was 1 cGy per monitor unit (MU) which agreed with the accelerator calibration.

With different motion ranges, the central point dose measurements and 4D dose calculations showed an agreement better than 3%. With a tumor motion range of 3 cm (Case 4), the measured central point dose for a 5 × 5 cm^2 ^field demonstrated a 27.5% ± 0.7% drop compared to the static phantom case, while the 4D dosimetry calculation showed a 25.0% ± 1.1% drop. With a motion range of 1.5 cm (Case 3), the central point dose was equivalent for both the phantom measurement and 4D dose calculation due to the fact that the central point was well covered by the treatment beams, given the relatively short motion range.

### Lung Tumor Treatment Plans

**Figure 2:**
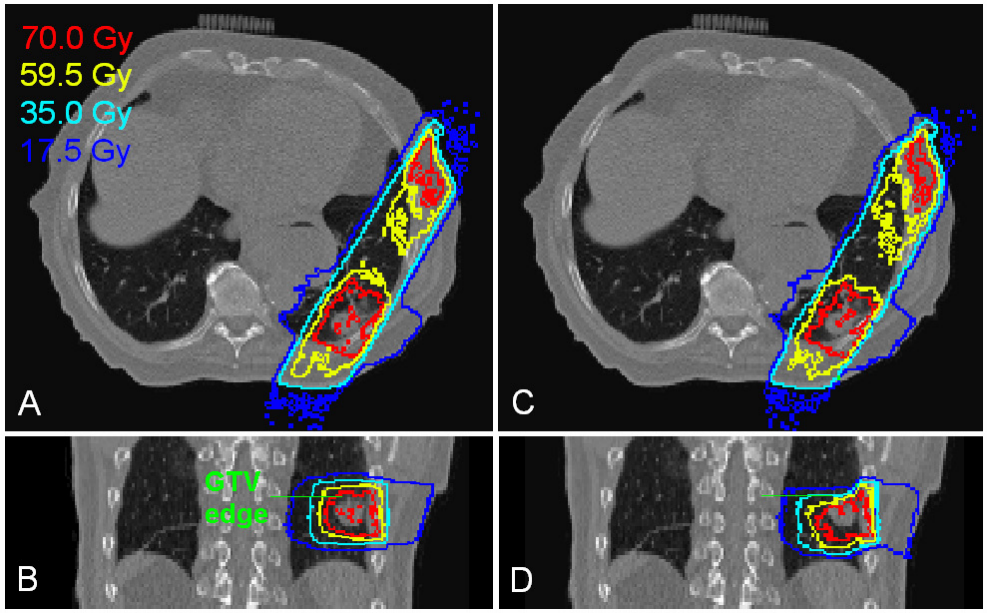
**Case 1 comparison of the Monte Carlo calculated static dose during the maximum inspiration phase (panels A and B), and the mapped static dose of the maximum expiration phase viewed on the maximum inspiration phase (panel C and D)**. The original static plan was optimized on the maximum inspiration phase. The coronal view of the mapped dose (panel D) shows that the tumor is closer to the upper isodose lines, which is expected because the tumor moves superiorly in the maximum expiration phase. The green lines on panel B and D indicate the GTV superior edge.

Figure 2 compares the Monte Carlo static dose distribution on the maximum inspiration phase (Figure 2A-B) with the static dose of the maximum expiration phase mapped onto the maximum inspiration phase image (Figure 2C-D). The distribution of the mapped dose is shifted inferiorly towards the diaphragm, and the tumor is closer to the superior aspect of the isodose distribution (Figure 2D). The reason for this is that in the diaphragm and tumor move upward in the maximum expiration phase while the beams remain fixed. Consequently, the dose distribution on the maximum expiration phase moves inferiorly relative to the diaphragm or tumor. Therefore, after the dose distribution is mapped onto the maximum inspiration phase, the isodose distribution skews inferiorly.

**Figure 3:**
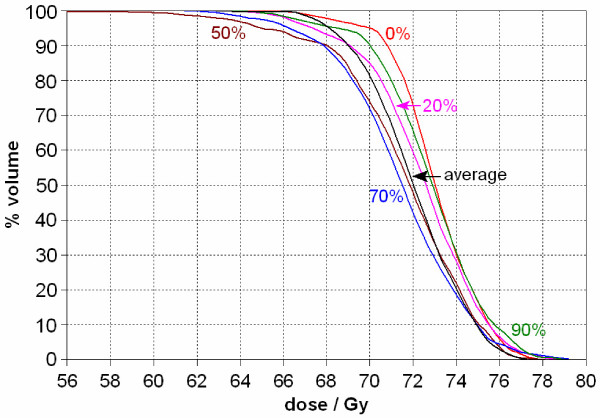
**Dose-volume histograms (DVH) of the gross tumor volume (GTV) from various static respiratory phases (0%, 20%, 50%, 70%, and 90%) as well as the 4D cumulative dose DVH (average) for Case 1**. In the static plan from the 0% phase, the GTV coverage at the prescribed dose of 70 Gy is about 95%, while it is 80% for the 4D cumulative dose.

Figure 3 shows a DVH of the GTV coverage at various phases of the respiratory cycle together with the 4D cumulative dose DVH. At the prescribed dose of 70 Gy, the static plan shows 95% GTV coverage in the maximum inspiration (0%) phase while the average dose plan only shows tumor coverage of 80%. The worst phase (50% or 70% in the figure) shows slightly better than 70% coverage of the GTV. In this example, the GTV moved about 1.5 cm in the SI direction. With a beam margin of 1 cm, tumor coverage was clearly reduced.

In general, the DVH of the 4D cumulative dose distribution from the mapped doses lies between the optimized static dose DVH at the maximum inspiration (0%) phase and the maximum expiration (50%) phase. However, at times, it can exceed or trail the curve for any individual phase. In Figure 3, at the low-dose portion of the curve, around 66 Gy, the volume covered by the average dose is higher than that for any of the static respiratory phases. Correspondingly, at the high dose tail (above 75 Gy), the average dose curve is lower than that for any individual respiratory phase. This behavior of the DVH curves in Figure 3 indicates that the 4D cumulative dose reduced the magnitude of hot/cold spots in individual static plans.

**Figure 4:**
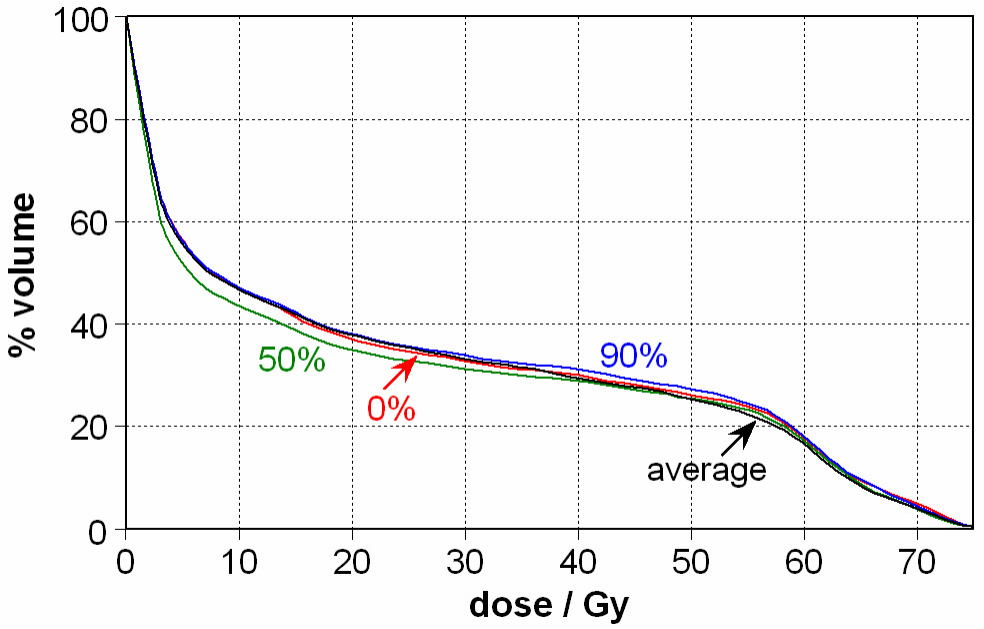
**Left lung DVHs from various static image sets (0%, 50%, 90%) and the 4D cumulative DVH (Case 1)**. For the 50% phase, the diaphragm started moving superiorly into the field, causing less lung being irradiated at this phase, thereby reducing the lung DVH.

When evaluating a treatment plan, one also needs to consider the DVH curves for the normal structures. In particular, different portions of lung move in and out of the treatment field, which causes the 4D cumulative lung DVH to vary from that for any given respiratory phase. This is evident in Figure 4.

**Figure 5:**
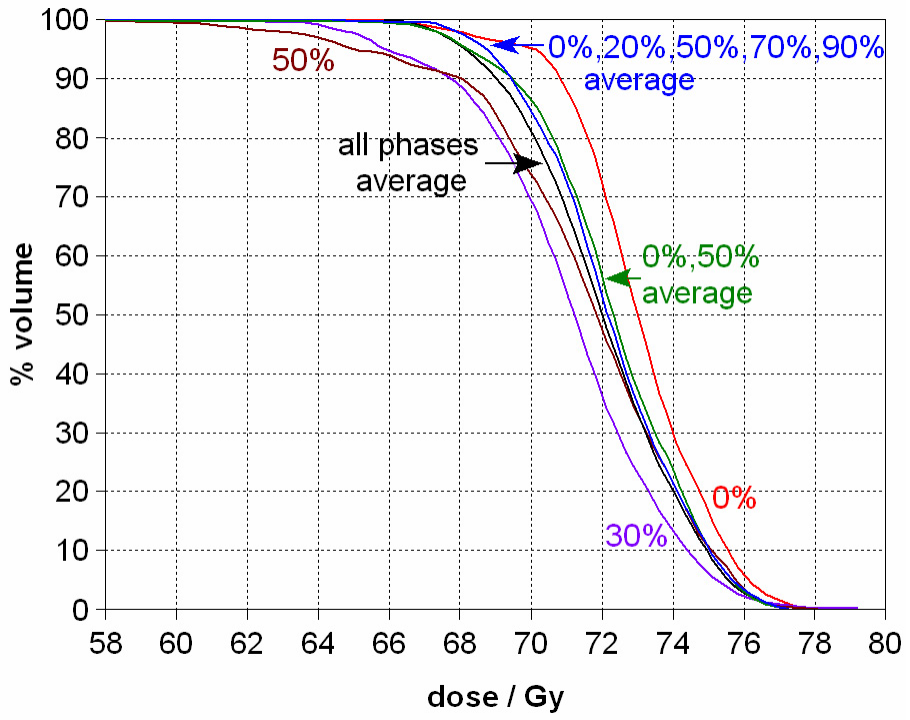
**Comparison of the 4D cumulative dose (average) DVH for the GTV when incorporating different numbers of respiratory phases into the calculations (Case 1)**. By incorporating additional phases, the accuracy of the dose calculation improved. However, the use of just two phases (0% and 50%, the maximum inspiration and maximum expiration respectively based on diaphragm motion) provides a reasonable approximation. The dose difference for the same volume coverage between each of the three averaged DVH curves is less than 0.5 Gy. The lowest GTV coverage occurred at 30%, which is shown in this figure too for reference.

We next investigated how many respiratory phases are necessary to include in the 4D calculations to reasonably estimate the average dose to the GTV as calculated when incorporating all ten respiratory phases. Figure 5 shows a comparison of several GTV DVH curves from Case 1, including curves from the extreme static phases and the lowest GTV coverage phase (30%) as references. The calculated average doses included the doses as mapped from a variable number of the respiratory phases, using deformable image registration, ranging from two (0% and 50%), to five (0%, 20%, 50%, 70% and 90%), to all the 10 phases. By observation, the inclusion of increasing numbers of respiratory phases in the 4D dose calculation improves agreement with the calculation derived from using all ten phases. However, considering that both Monte Carlo simulation and deformable image registration are time consuming calculations, the DVH of the cumulative dose using just the two extreme phases is a reasonable representation of the average derived when incorporating all ten phases.

**Figure 6:**
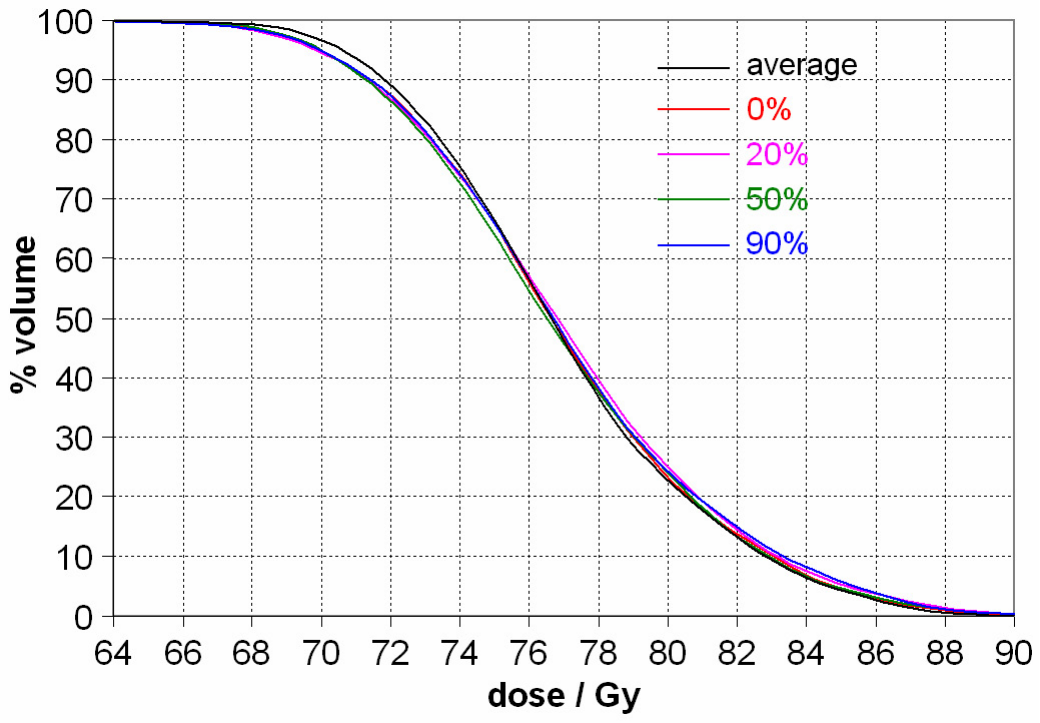
**GTV DVH curves from various static respiratory phases (0%, 20%, 50%, and 90%) and the 4D cumulative dose for Case 2**. The GTV was large (159.1 cm^3^) in relation to the tumor motion (1 cm). This is in contrast to Case 1, which had a similar range of tumor motion, but for a tumor which measured only 12.5 cm^3^. Consequently, the DVH curve for the average dose does not differ much from the static DVH curves (Figure 3).

In Case 2, the GTV motion is about 1 cm, but the DVH variation is much smaller than that in Case 1 even with a block margin of 0.5 cm across the GTV (Figure 6). This can be explained by the fact that the GTV is much larger in Case 2 (159.1 cm^3^) than in Case 1 (12.5 cm^3^). This translates into a much smaller percentage volume change for Case 2 when compared to Case 1.

## Discussion

In this study, discrepancy between a point dose measurement in a moving phantom and the calculated 4D cumulative dose was less than 3%. The variance is multifactorial, representing a combination of errors from Monte Carlo simulation, image registration, and phantom measurements.

In the Monte Carlo simulations, the statistical uncertainties in the high dose regions, such as the GTV, are below 1%. Other error sources include electron source parameters, linear accelerator geometry and materials. Any discrepancies of these items between simulation and reality could introduce variability between calculations and measurements. As shown previously, these differences were within 2% for most cases in our study.

Errors in image registration can also affect the calculated dose. There are three root causes for errors in image registration. Artifacts in the 4D CTs, the aperture effect [18], and the inherited occlusion problem [19] all introduce potential sources for error in image registration. In our experience, 4D CT artifacts are the major contributing factor to errors in image registration. The 4D CT artifacts are caused by residual motion in each respiration phase which smears details in 4D CT images. Since accurate optical flow registration depends upon clarity of the details in each image, any degradation in image quality can impact the quality of registration.

The aperture effect is introduced in regions of flat intensity within the images. When there is no variation in intensity within a region, the voxel-to-voxel correspondence becomes vague. Thus the registration may have larger errors in low contrast regions. For human CT data, detailed anatomic structures, such as veins, help reduce the aperture effect. Our prior research has shown that the average magnitude of this error is smaller than an image voxel size in the thoracic regions [20]. Another study by Zhong *et al *[21] showed that the average error in lungs by Demons, another deformable image registration algorithm that is similar to optical flow, was around 0.7 mm, but larger in the low gradient prostate region.

Occlusion may cause motion discontinuity in other image registration applications, such as daily patient CT registration when rectal filling varies. For 4D CT images, occlusion is not a problem since there is no topological change between the respiratory phase images.

The Monte Carlo method applied in this study is a classical full Monte Carlo method. The calculation time was long for each case. In recent a few years, various techniques have helped in increasing the computational efficiency of Monte Carlo simulation and reducing its calculation time [3,22-24]. Using multiple source models instead of simulating phase-space files would also reduce calculation time significantly [24]. By applying these modifications, some simpler and faster Monte Carlo methods have already been implemented in commercial treatment planning systems or demonstrated to be reasonable for clinical application [25-27]. With faster computers and high efficient Monte Carlo algorithms, multi-phase Monte Carlo dose calculations have been demonstrated feasible for clinical applications [27]. If fewer phases are used for 4D dose calculations, the work load is also correspondingly reduced. Another way to further reduce the computation time is to lower the simulation histories in each respiration phase. With a higher statistical uncertainty in each respiration phase, the statistical uncertainty of the 4D cumulative dose remains at an acceptable level [8]. The 4D Monte Carlo dose calculation can be reduced to a single calculation on the average CT if the simplified 4D dose accumulation method proposed by Glide-Hurst *et al *[28] is applied.

In our 4D test cases, the method noticeably altered the dose calculation compared to static plans only when the tumor was small and the respiratory motion was comparatively large.

Vinogradskiy *et al *[29] demonstrated by measurement that 4D dose calculations provided greater accuracy than 3D dose calculations in heterogeneous dose regions. Rosu *et al *[30] studied how many phases are needed in 4D cumulative dose calculation for various clinical end points and concluded that results using only two extreme phases in 4D cumulative dose calculation agreed well with those of full inclusion for the 4 cases studied. This study confirmed their conclusion with Monte Carlo calculations.

The treatment plans generated for this study were not intended for clinical use. The phase for the original plan was randomly picked between the two extreme phases and the isocenter was placed on the GTV center of the corresponding phase. The margins in the plans were purposely set small compared to the motion ranges so that target volume coverage loss, thus DVH variation of the target volume versus respiratory phase, was more pronounced. The conditions used in our study tended to exaggerate coverage loss and hence was more adverse against the above conclusion. The conclusion is thus deemed more confident when applied to real clinical cases which are usually with better coverage. However, due to limited number of cases studied, this conclusion should not be applied to cases of larger or irregular motions. When large motion is reduced to be within certain range (< 1 cm) by applying a motion-reducing technique, such as abdominal compression which is often used in stereotactic lung treatments, this conclusion should apply as long as the beam margins are large enough for the motion ranges.

Monte Carlo methodology provides more accurate dose calculation across an inhomogeneous substrate such as the lung [31]. For some extrathoracic sites, such as the abdomen, respiratory motion of tumors and normal structures is not insignificant [32]. Therefore, 4D dose calculations might also prove useful in the treatment of abdominal tumors. When lung or any other significant inhomogeneous substrate is not involved in treatment volumes, Monte Carlo methods may be replaced by other faster dose calculation algorithms in 4D dose calculations with an acceptable accuracy.

## Conclusions

With the combination of Monte Carlo simulation and the optical flow method, 4D dosimetry is proved accurate based on point-dose measurement in a moving phantom. Monte Carlo 4D dose calculation would provide a planned dose distribution that is closer to the delivered dose than a static plan does, especially when dose variation is large between respiratory phases. Based on the cases studied, large dose variation between respiratory phases is more likely for small tumor volumes with relatively large motion. The inclusion of only two extreme respiratory phases in 4D cumulative dose calculation would be a reasonable approximation to all-phase inclusion for cases similar to the ones studied.

## Competing interests

The authors declare that they have no competing interests.

## Authors' contributions

TC: performed most data measurement and calculation; contributed in data analysis; carried out programming; participated draft of manuscript. JA: participated data acquisition; contributed in draft of manuscript. TD: provided patient contours and treatment prescriptions; guided treatment plans; contributed in draft of manuscript. TH: coordinated the collaboration; contributed in data analysis and draft of manuscript. GZ: contributed the frame work of the project, participated data analysis; contributed in draft of manuscript; supervised the project. All authors read and approved the final manuscript.

## References

[B1] Rogers DWO Faddegon BA Ding GX Ma C-M We J Mackie TR BEAM: A Monte Carlo code to simulate radiotherapy treatment units Med Phys 1995 22 503 524 10.1118/1.597552 7643786

[B2] Verhaegen F Seuntjens J Monte Carlo Modelling of external radiotherapy photon beams. Phys Med Biol 2003 48 R107 R164 14653555 10.1088/0031-9155/48/21/r01

[B3] Fippel M Fast Monte Carlo dose calculation for photon beams based on the VMC electron algorithm Med Phys 1999 26 1466 1475 10.1118/1.598676 10501045

[B4] Fippel M Efficient particle transport simulation through beam modulating devices for Monte Carlo treatment planning Med Phys 2004 31 1235 1242 10.1118/1.1710734 15191314

[B5] Ma C-M Li JS Pawlicki T A Monte Carlo dose calculation tool for radiotherapy treatment planning Phys Med Biol 2002 47 1671 1689 10.1088/0031-9155/47/10/305 12069086

[B6] Wang L Chui C-S Lovelock M A patient-specific Monte Carlo dose-calculation method for photon beams Med Phys 1998 25 867 878 10.1118/1.598262 9650174

[B7] Yu CX Jaffray DA Wong JW The effects of intra-fraction organ motion on the delivery of dynamic intensity modulation Phys Med Biol 1998 43 91 104 10.1088/0031-9155/43/1/006 9483625

[B8] Keall PJ Siebers JV Joshi S Mohan R Monte Carlo as a four-dimensional radiotherapy treatment-planning tool to account for respiratory motion Phys Med Biol 2004 49 3639 3648 10.1088/0031-9155/49/16/011 15446794

[B9] Paganetti H Jiang H Adams J Chen G Rietzel E Monte Carlo simulations with time-dependent geometries to investigate effects of organ motion with high temporal resolution Int J Radiat Oncol Biol Phys 2004 60 942 950 10.1016/j.ijrobp.2004.06.024 15465213

[B10] Guerrero T Zhang G Segars W Elastic image mapping for 4-D dose estimation in thoracic radiotherapy Radiat Protection Dosimetry 2005 115 497 502 10.1093/rpd/nci225 16381774

[B11] Zhang G Huang T-C Forster K Dose mapping: validation in 4D dosimetry with measurements and application in radiotherapy follow-up evaluation Comp Meth Prog in Biomed 2008 90 25 37 10.1016/j.cmpb.2007.11.015 18178288

[B12] Kawrakow I Accurate condensed history Monte Carlo simulation of electron transport. I. EGSnrc, the new EGS4 version Med Phys 2000 27 485 498 10.1118/1.598917 10757601

[B13] Kawrakow I Walters BRB Efficient photon beam dose calculations using DOSXYZnrc with BEAMnrc Med Phys 2006 33 3046 3056 10.1118/1.2219778 16964882

[B14] Ma C-M Reckwerdt P Holmes M Rogers DWO Geiser B DOSXYZ Users Manual NRC Report 1995 Ottawa, Canada: National Research Council Canada

[B15] da Rosa L Cardoso S Campos L Alves V Batista D Facure A Percentage depth dose evaluation in heterogeneous media using thermoluminescent dosimetry J Appl Clin Med Phy 2010 11 117 127 10.1120/jacmp.v11i1.2947 PMC5719771 20160687

[B16] Künzler T Fotina I Stock M Georg D Experimental verification of a commercial Monte Carlo-based dose calculation module for high-energy photon beams Phys Med Biol 2009 54 7363 7377 10.1088/0031-9155/54/24/008 19934489

[B17] Horn BKP Schunck BG Determining optical flow Artif Intell 1981 17 185 203 10.1016/0004-3702(81)90024-2

[B18] Beauchemin SS Barron JL The computation of optical flow ACM Computing Surveys (CSUR) 1995 27 433 466 10.1145/212094.212141

[B19] Geman S Geman D Stochastic relaxation, gibbs distributions, and the Bayesian restoration of images IEEE Trans Pattern Analysis Machine Intell 1984 6 721 741 10.1109/TPAMI.1984.4767596 22499653

[B20] Guerrero T Zhang G Huang T-C Lin K-P Intrathoracic tumour motion estimation from CT imaging using the 3D optical flow method Phys Med Biol 2004 49 4147 4161 10.1088/0031-9155/49/17/022 15470929

[B21] Zhong H Kim J Chetty IJ Analysis of deformable image registration accuracy using computational modeling Med Phys 2010 37 970 979 10.1118/1.3302141 20384233 PMC3188658

[B22] Bielajew AF Rogers DWO Jenkins TM, Nelson WR, Rindi A Variance-reduction techniques Int. School of Radiation Damage and Protection, Eighth Course: Monte Carlo Transport of Electrons and Photons below 50 MeV 1988 New York: Plenum 407 419

[B23] Fippel M Haryanto F Dohm O Nüsslin F Kriesen S A virtual photon energy fluence model for Monte Carlo dose calculation Med Phys 2003 30 10.1118/1.1543152 12674229

[B24] Ma C-M Mok E Kapur A Clinical implementation of a Monte Carlo treatment planning system Med Phys 1999 26 2133 2143 10.1118/1.598729 10535630

[B25] Sempau J Wilderman SJ Bielajew AF DPM, a fast, accurate Monte Carlo code optimized for photon and electron radiotherapy treatment planning dose calculations Phys Med Biol 2000 45 2263 2291 10.1088/0031-9155/45/8/315 10958194

[B26] Siebers JV Keall PJ Kim JO Mohan R A method for photon beam Monte Carlo multileaf collimator particle transport Phys Med Biol 2002 47 3225 3249 10.1088/0031-9155/47/17/312 12361220

[B27] Söhn M Weinmann M Alber M Intensity-Modulated Radiotherapy Optimization in a Quasi-Periodically Deforming Patient Model Int J Radiat Oncol Biol Phys 2009 75 906 914 19747782 10.1016/j.ijrobp.2009.04.016

[B28] Glide-Hurst CK Hugo GD Liang J Yan D A simplified method of four-dimensional dose accumulation using the mean patient density representation Med Phys 2008 35 5269 5277 10.1118/1.3002304 19175086 PMC2673609

[B29] Vinogradskiy YY Balter P David SF Alvarez PE White RA Starkschall G Comparing the accuracy of four-dimensional photon dose calculations with three-dimensional calculations using moving and deforming phantoms Medical Physics 2009 36 5000 5006 10.1118/1.3238482 19994509

[B30] Rosu M Balter JM Chetty IJ How extensive of a 4D dataset is needed to estimate cumulative dose distribution plan evaluation metrics in conformal lung therapy? Med Phys 2007 34 233 245 10.1118/1.2400624 17278509

[B31] DeMarco JJ Solberg TD Smathers JB A CT-based Monte Carlo simulation tool for dosimetry planning and analysis Med Phys 1998 25 1 11 10.1118/1.598167 9472820

[B32] Feng M Balter JM Normolle DP Characterization of pancreatic tumor motion using 4D MRI: surrogates for tumor position should be used with caution Int J Radiat Oncol Biol Phys 2007 69 S3 S4 10.1016/j.ijrobp.2009.02.003 PMC2691867 19395190

